# Midwives’ experiences regarding recordkeeping during intrapartum care in Limpopo Province healthcare facilities

**DOI:** 10.4102/curationis.v47i1.2594

**Published:** 2024-10-25

**Authors:** Phogole C. Maesela, Johanna M. Mathibe-Neke

**Affiliations:** 1 Department of Health Studies, School of Social Sciences, College of Human Sciences, University of South Africa, Pretoria, South Africa

**Keywords:** experience, intrapartum care, midwife, quality, recordkeeping

## Abstract

**Background:**

South Africa has experienced an increase in litigations because of poor recordkeeping. The quality of maternal healthcare necessitates quality recordkeeping. All midwives’ interventions should be documented in the maternity case record as an instrument to highlight the quality of intrapartum care offered.

**Objectives:**

The purpose of the study was to determine and describe the experiences of midwives regarding recordkeeping during intrapartum care in Limpopo province and to make recommendations to improve recordkeeping.

**Method:**

A qualitative, explorative and descriptive design was adopted. Midwives were selected purposively to participate in focus group discussions. Data were thematically analysed with the help of the independent transcriptionist and coder.

**Results:**

The findings revealed the themes: perceptions of midwives regarding recordkeeping and the challenges and enablers that influence recordkeeping during intrapartum care.

**Conclusion:**

Quality recordkeeping requires timely, detailed, comprehensive and accurate recording. The study recommended the availability of updated guidelines, in-service training, monitoring and evaluation of recordkeeping, peer review, record auditing, proper time management among midwives and appointment of staff in line with the staffing needs of the unit to enhance recordkeeping.

**Contribution:**

Quality recordkeeping has a positive impact on the provision of quality healthcare to mothers during intrapartum care and reduces litigations related to maternity cases.

## Introduction

### Background

South Africa’s National Guidelines for Maternity Care (Department of Health [DoH] 2016) recommended that all hospitals should use a standardised maternity case record to document prenatal, intrapartum and postpartum care and treatment. Department of Health (2016) further recommends that the healthcare system should provide quality recordkeeping. Moreover, the Situation Background Assessment recommendation (SBAR) referral letter outlines the specifications for the format and content of the maternity case records (DoH 2016).

A study by Sibiya, Cele and Ngxongo (2015), conducted in KwaZulu-Natal, revealed that midwives perceive the new maternity case record as not user-friendly.

A lack of time to complete records, increased patient numbers and a shortage of recording materials were found to be the challenges for nurses working in public hospitals in Limpopo province, in South Africa, as reported by Mutshatshi et al. (2018). However, a study conducted in the Vhembe District concluded that patients’ records were incomplete, and some information was never recorded, despite the availability of recording forms (Shihundla, Lebese & Maputle 2016).

Marutha and Ngoepe (2017) conducted a study in Limpopo province on the role of medical records in the provision of public healthcare services and found out that most nursing staff have not received any formal training regarding the policies, procedures, norms and standards for managing records, hence nurses were unable to assist patients or treat them immediately.

Taiye (2015) states that documentation is an integral part of nursing and midwifery practice because effective communication among health professionals is vital to the quality of client care. The level of care provided by midwives is determined by quality recordkeeping that includes documentation during intrapartum care. Griffith (2016) highlights that the main aim of keeping records is to have a record of the interventions offered to the mother and child. Research that compared world standards with those of Iran revealed that nurses exhibit more desirable qualities regarding adherence to documentation principles, and the documents were evaluated as accurate, professional and concise (Vafaei et al. 2018).

A study conducted in Bloemfontein, South Africa, by Brits et al. (2020), indicated that more than 70% of the partograms scored more than 75% for completion. However, critical components that influence maternal and foetal death such as identification of foetal distress, maternal well-being and progress of labour were not completed.

### Purpose of the study

The study aimed to determine and describe midwives’ experiences regarding recordkeeping during intrapartum care in Sekhukhune District, Limpopo province.

## Research methods and design

### Study design

A qualitative, interpretative descriptive design was applied in this study as a naturalistic approach and aimed to understand the phenomena through each participant’s perspective in their natural setting (Bradshaw, Atkinson & Doody 2017; Duda, Warburton & Black 2020; Hazzi & Maldaon 2015; Polit & Beck 2022).

### Research setting

The study was conducted in six selected hospitals located in four local Municipalities in Sekhukhune District, Limpopo province.

### Population and sampling

The population of this study as guided by Polit and Beck (2022) were midwives working in the maternity unit for 6 months or more in these seven selected hospitals. Purposive sampling was used to recruit midwives from the six selected hospitals in the Limpopo province.

### Data collection

Six focus group discussions were conducted with 29 midwives who consented to participate in the study. Written consent was obtained from each participant including an audio recording of their responses (Schneider & Fuller 2018; Shah et al. 2021). The participation in this study was voluntary (Nieswiadomy & Bailey 2018). Kallio et al. (2016:2954) advised that focus group discussions should use the semi-structured interview guide. A semi-structured interview guide was used as the data collection tool (Marshall & Rossman 2016). The questions were formulated in English as participants were midwives and advanced midwives. The discussions occurred in private rooms to ensure privacy. The focus group discussions were audio-recorded and lasted between 30 min and 45 min. As per World Health Organization’s (WHO’s 2020) coronavirus disease 2019 (COVID-19) guidelines, participants were instructed to wear their masks, not to touch their eyes, clean their hands with water and soap or an alcohol-based hand rub before and after the discussions and to follow proper coughing etiquettes, which are to cover the nose and mouth with the bent elbow or a tissue.

### Data analysis

For this study, as guided by McEvoy, Tierney and MacFarlane (2019), Mihas (2019), Fellows and Liu (2021) and Taherdoost (2022), data were analysed through coding, by applying Tech’s data analysis steps, where data were organised into themes and sub-themes. The independent qualitative researcher assisted in confirming the themes and sub-themes that emerged.

### Trustworthiness

Credibility refers to whether the research participants validate the outcomes and whether the findings make sense to other researchers (Willig & Rogers 2017). In this study, credibility was achieved through prolonged face-to-face engagement with six focus group discussions with midwives where audio tapes and field notes were kept as part of the audit trail. Transferability was achieved through the provision of a detailed description of the study’s design, setting and participants to allow the reader to draw conclusions about the transferability of the results to other settings, as guided by Olson et al. (2016). Conformability was achieved by compiling a full record of the data collection and analysis strategies used (Gray, Grove & Sutherland 2017). Dependability was enhanced by outlining the detailed design, methodology, data collection using the voice recorder and taking of field notes (Forero et al. 2018). According to Lazard and McAvoy (2017), reflexivity is a form of critical thinking that involves addressing the issues of identity and positionality by making the researcher’s assumptions explicit and finding strategies to respond to the assumptions. Reflexibility of this study was achieved by detailing the process followed from the study orientation to the conclusion of the study.

### Ethical consideration

Ethical approval was obtained from the University of South Africa (44439121_CREC_CHS_2020). Permission to contact the study was sought from the Limpopo DoH (LP-2020-09- 011) and Sekhukhune District Health (Ref: S2/2/3) where the healthcare facilities are located. All participants signed a consent and confidentiality binding form. The ethical principles of voluntary participation, justice and beneficence were upheld throughout the study.

## Results

Twenty-nine midwives participated in the six focus group discussions from the selected healthcare facilities. The selected healthcare facilities were coded as DH1 (District Hospital 1), DH2, DH3, DH4, DH5 and RH1 (Regional Hospital 1). The number and categories of participants are illustrated in Table 1.

**TABLE 1:** Code, categories and number of participants per focus group discussion.

Healthcare facility	Participants’ categories	Number of participants
DH1	Midwives	6
DH2	Midwives	4
DH3	Midwives	5
DH4	Midwives	3
DH5	Midwives	6
RH 1	Midwives	5

*Source:* Maesela, P.C., 2023, ‘The quality of recordkeeping during intrapartum care in Limpopo province: A mixed method analysis’, PhD thesis, University of South Africa, https://uir.unisa.ac.za/handle/10500/31212

DH, district hospital; RH, regional hospital.

Three major themes and sub-themes that emerged from the data are reflected in Table 2.

**TABLE 2:** Themes and sub-themes.

Themes	Sub-themes
1 Perceptions of midwives regarding recordkeeping	1.1 Availability of guidelines 1.2 Good recordkeeping practices 1.3 Fear of litigation
2 Enablers to the implementation of recordkeeping	2.1 Importance of staff teamwork 2.2 Management support
3 Challenges in recordkeeping	3.1 Inadequate time 3.2 Impact of shortage of staff (midwives) 3.3 A lack of proper skills

*Source:* Maesela, P.C., 2023, ‘The quality of recordkeeping during intrapartum care in Limpopo province: A mixed method analysis’, PhD thesis, University of South Africa, https://uir.unisa.ac.za/handle/10500/31212

## Discussion of the findings

These findings are based on data collected and analysed from six focus group discussions. Three major themes that emerged are discussed in this section.

### Theme 1: Perceptions of midwives regarding recordkeeping

During the focus group discussions, midwives shared various perceptions regarding recordkeeping in the selected healthcare facilities.

#### Sub-theme 1.1: Availability of guidelines

Midwives expressed different perceptions regarding the availability of recordkeeping guidelines.

Most of the participants highlighted that recordkeeping guidelines are received by heads of the selected healthcare facilities with limited access:

‘The facility has the guidelines that are allocated to the maternity units. The district supply to our facility with the guidelines is always in line with the hospital need.’ (Participant 1, DH3, Midwives)

‘As midwives, we worry about the availability of policies and guidelines. It is shocking that the hospital has got policies and guidelines about recordkeeping, but no manager takes us into confident that we know about them.’ (Participant 2, DH3, Midwives)

Other participants indicated the unavailability of guidelines:

‘There are neither recordkeeping guidelines, policies, standard operating procedures [*SOPS*] nor protocols in our units to be used as a source of reference during maternity care of the mothers. It is for that reason that most of the time midwives are not sure of what we should do as they run around.’ (Participant 5, DH5, Midwives)

The research findings regarding the availability of recordkeeping guidelines by Mutshatshi et al. (2018) in Limpopo province highlighted the challenges in the dissemination of guidelines to different hospitals.

#### Sub-theme 1.2: Good recordkeeping practices

Midwives’ perceptions of good recordkeeping practice during intrapartum were discussed as:

‘In this health establishment, we try to keep records of all the activities that happened during the maternal care including intrapartum care. When gaps are found during record auditing and peer review, they are addressed best to record. It must be noted that recordkeeping is important.’ (Participant 3, DH2, Midwives)

‘In this facility, management, the midwives emphasise good clinical practice in recordkeeping as a daily integral part of rendering quality patient care including the completion of the maternal case record.’ (Participant 4, DH1, Midwives)

Garba and Yahaya (2018) highlighted recordkeeping as a vital instrument in the practice of midwifery. Moreover, the study concluded that recordkeeping is essential to preserve and use patient information effectively.

#### Sub-theme 1.3: Fear of litigation

The participants expressed their feelings on the shortage and retention of staff at their facilities as one of the contributory factors towards poor recordkeeping and further highlighted that midwives are exposed to a risk of litigation because of poor recordkeeping:

‘As a midwife, I am afraid to commit and decide and act by recording the decision in the maternity case record as I may land in the regulatory body [*SANC*] and be charged with acts or omission accordingly. A charge from the SANC can be a base where the midwife is litigated.’ (Participant 4, RH1, Midwives)

‘Midwifery is a risky nursing profession. Midwives encounter pressure from patients, family members, hospital management and the community hospital. New community health nurses resolved not to practice midwifery due to the inflating litigations related to midwifery cases. Dedicated midwives are leaving midwifery sector due to the litigation that may lead them to lose their profession.’ (Participant 1, DH3, Midwives)

Participants concluded that fear of litigation leads to continuous unresolved conflicts between the midwives. Magqadiyane (2020) conducted a qualitative study on midwives’ experiences in maternal health litigations to determine perspectives from a rural district hospital in South Africa and found out that despite the meetings held with state attorneys, most of these cases are not wined by the state attorneys because of multiple unresolved issues, the litigations continue happening.

### Theme 2: Enablers to the implementation of recordkeeping

The participant’s response highlighted staff teamwork and management support as the enablers to improve recordkeeping during intrapartum care.

#### Sub-theme 2.1: Importance of staff teamwork

Staff teamwork contributes to improved recordkeeping during intrapartum care:

‘Midwives are saved by teamwork reminding one another to record in the maternity case recordkeeping of the patient safety incidents during investigations. Midwives collaborate and work together to benefit the patients and families.’ (Participant 1, DH2, Midwives)

‘The members of the midwifery team in our hospital may also have disagreements over the matters related to quality recordkeeping but it is for the benefit of learning.’ (Participant 5, DH3, Midwives)

The findings of this study recommended that the midwives and advanced midwives should work together in all the processes of managing the woman in labour including intrapartum care (Weiseth et al. 2022).

#### Sub-theme 2.2: Management support

The response from midwives highlighted minimal support from management related to matters of recordkeeping during intrapartum care:

‘There is a poor relationship between the management and the staff which is caused by lack of empowerment from the hospital management regarding recordkeeping, The operational manager and the nursing service manager are new without vast experience in maternity-related challenges.’ (Participants 5, DH1, Midwives)

‘Managers expect the midwives to solve the strategic problems such as availing sufficient numbers of maternity case records. Midwives are expected to resolve operational problems such as recordkeeping changes.’ (Participant 4, RH1, Midwives)

The findings of this study were supported by the study conducted by Kanyabwira (2024) on the impact of records management who concluded that management’s role to support, empower and distribute information on recordkeeping is vital. The study conducted by Rawah and Banakhar (2022) in Saudi Arabia about the relationship between empowerment and organisational commitment from a nurse’s perspective in the Ministry of Health hospitals concurred with the findings of this study by revealing the poor relationship between the management and the staff, which is caused by a lack of empowerment of nurses from the hospital management. The recommendations were made to the management to bridge the gap to support the midwives (De Leo et al. 2019). The management of the hospitals should host training sessions, seminars, conferences and symposiums on recordkeeping to assist the midwives (Mutshatshi et al. 2018). The findings of this study were further corroborated by Hastings-Tolsma (2021) who concluded that there was widespread belief that management support for midwives is often lacking.

### Theme 3: Challenges in recordkeeping

The participants reported insufficient time to record in the maternity case record and a shortage of skilful and experienced midwives as challenges associated with recordkeeping. The participants’ responses are discussed next

#### Sub-theme 3.1: Inadequate time

The participants justified their lack of time to document the maternity case records, which led to retrospective recording as follows:

‘The variables in the maternity case records are many and I usually take time to complete the maternity case record after the delivery. The writing is too much and sometimes is disturbed by admissions and other deliveries in the unit. We experience more deliveries and we do not keep up with the principles of recordkeeping hence records are completed retrospectively.’ (Participant 2, RH1, Midwives)

‘Once upon a time in this hospital, I was on duty with only one enrolled nursing assistant with eight women in labour and three were fully dilated, three were in an active labour stage and two were in the latent labour stage. I decided to ignore the principles of recordkeeping and concentrate on the delivery of these women.’ (Participant 1, DH 2, Midwives)

Muyakui, Nuuyoma and Amakugo (2019) conducted a qualitative study in Namibia on recordkeeping reflecting on the challenges of recording, which had more to do with clinical settings related to practical and theory gaps, hospital-related and hospital staff-related challenges.

Another study by Fadana and Vember (2021) found that undergraduate nursing students in selected clinical settings found the challenges of recordkeeping to be a time waster as the participants felt they spent more time recording than actually attending to the patients. The research participants highlighted the challenge of spending more time filling in all the forms related to the maternity process. During the assessment of patients, students felt they spent a lot of time documenting rather than focussing on patient’s observations.

#### Sub-theme 3.2: Impact of shortage of staff (midwives)

The participants of this study depicted the gaps related to recordkeeping, which were reported as follows:

‘Shortage of midwives makes midwives to work long hours without breaks, as the midwife, I feel stressed and exhausted due to a hospital shortage of staff, I will just visit the doctor and consult and take sick leave days and I know that the action will deepen shortage and increase stress level of the midwives in the unit.’ (Participant 5, DH3, Midwives)

‘Staffing is always posing a threat to our operations; the hospital must prioritise the recruitment of experienced midwives.’ (Participant 3, RH1, Midwives)

The negative impact of a shortage of staff is supported by Suhaimi, Mulud and Sharoni (2021) who concluded that midwives find it challenging to maintain high standards of recordkeeping throughout intrapartum care because of a shortage of midwives in the maternity wards. Manyisa and Van Aswegen (2017:36) are of the opinion that the shortage of midwives and the lack of resources are all factors that affect working conditions and lead to exhaustion among midwives.

#### Sub-theme 3.3: A lack of proper skills

The study respondents detailed that a lack of skills in midwifery particularly in the labour ward may pose a challenge in implementing the recordkeeping guidelines:

‘Most of the midwives in our hospital have only basic nursing with midwifery qualification. The experience counts a lot in plotting the partograph which is still a challenge.’ (Participant 1, DH4, Midwives)

‘I have realised that sometimes, inexperienced midwives are delegated to be in charge of labour unit at night when all managers are not in the hospital, the situation may be risky if more than two pregnant can come whilst in an active stage of labour.’ (Participant 4, DH5, Midwives)

‘We could hardly be given a chance to improve ourselves through skill development act to advance our skill by attending the trainings but, only the top and senior manager are receiving such opportunities.’ (Participant 1, DH1, Midwives)

A lack of skill and experience is supported by Kleinpell and Zimmerman (2017), who found a lack of skills as the challenge to implement clinical guidelines effectively.

## Recommendations of the study

Recommendations to enhance recordkeeping were developed based on the findings.

### Availability of the updated recordkeeping guidelines

Management to ensure the availability of guidelines to enhance quality recordkeeping.

According to Stokes et al. (2016), an effective implementation strategy requires flawless guidelines distribution and availability. The findings of this research determined that managers should take decisional and managerial roles by supporting the staff to implement the guidelines and work together as teams to implement the good principles of recordkeeping.

### In-service training

According to the *Nursing Act 33 (2005)*, midwives in the maternity units should develop an in-service training plan to discuss intrapartum care variables in the maternity case records and other clinical in-patient records. The in-service training will serve as empowerment for the midwives to apply good recordkeeping principles. Mosweu and Rakemana (2020) concluded that appropriate training is another factor that hinders good records management practices in Africa. Furthermore, the study recommended that senior midwives should prioritise in-service training for junior midwives on the recording of maternity case records.

### Monitoring and evaluation of nurses on recordkeeping issues

The participants of the focus group discussions recommended that there should be a system such as participation of operational, area and nursing service managers involved in enhancing recordkeeping.

Tamir, Geda and Mengistie (2021) discovered that nurses who were motivated by their supervisors regarding documentation activities were more likely to practice documentation when compared to nurses who were never motivated. Furthermore, it was discovered that attitudes towards nursing documentation and in-service training were positively associated with self-reported documentation practices. Specifically, the standards of nursing documentation, familiarity with operational standards, motivation from supervisors, nurse-to-patient ratio and age of nurses all showed a statistically significant positive correlation (Tamir et al. 2021:461).

### Peer review for record auditing

Peer review and record auditing of maternity case records enhance recordkeeping and the quality of maternal healthcare. The study’s findings were supported by Lalloo, Demou and Macdonald (2015), who examined the impact of peer review audits on occupational health report quality. The peer review process not only improved the standard of occupational health reports but was also associated with a reduction in clinical complaints related to those reports.

### Proper time management among midwives

It was also recommended that healthcare facilities arrange time management and in-service training workshops for midwives to help them effectively manage their time and enhance recordkeeping during intrapartum care.

### Appointment of staff in line with the staffing needs

The participants recommended that midwives should be appointed in line with the staffing needs and priorities. The study findings were corroborated by research conducted by Matlala and Lumadi (2019) who recommended that midwifery management should address midwives’ fear of litigations, negative attitudes, skills deficit, shortage of skilled midwives and time management workshops for midwives to enhance recordkeeping during intrapartum care.

### Limitations of the study

The experience regarding recordkeeping was only of the midwives working in selected public hospitals in the Sekhukhune District of Limpopo province. One healthcare facility including their midwives withdrew from participating in the focus group because of the COVID-19 situation.

## Conclusion

The study’s findings have implications to enhance quality recordkeeping that impacts the provision of quality healthcare to mothers during intrapartum care. Furthermore, the findings can be used to develop recordkeeping guidelines for maternity.
